# Reablement in community-dwelling older adults: a cost-effectiveness analysis alongside a randomized controlled trial

**DOI:** 10.1186/s13561-016-0092-8

**Published:** 2016-05-10

**Authors:** Egil Kjerstad, Hanne Kristin Tuntland

**Affiliations:** 1 Uni Research Rokkan Centre, P.O. Box 7810, Bergen, 5020 Norway; 2 Centre for Care Research Western Norway and Department of Occupational Therapy, Physiotherapy and Radiography, Bergen University College, P.O. Box 7030, Bergen, Norway

**Keywords:** Rehabilitation, Randomized controlled trial, Economic evaluation, Home care services

## Abstract

**Background:**

In the face of a growing number of older adults in the population, policy-makers in high-income countries are seeking new ways to reduce the expected growth in long-term care expenditure. Research shows that disability is an important determinant of long-term care utilization. In this context, reablement has received increased attention. Reablement is a form of home-based rehabilitation, which focuses on improving independent functioning in daily activities perceived as important by the older adult.

**Objective:**

To evaluate the cost-effectiveness of reablement.

**Methods:**

The economic evaluation is based on data from a randomized controlled trial in which all participants were assessed at baseline and after 3 and 9 months. The intervention group participated in reablement, while the control group received usual care. The Canadian Occupational Performance Measure (COPM) was used to measure self-perceived activity performance and satisfaction with performance. Cost data were based on daily registrations of usage of home-based care personnel during a period of 9 months.

**Results:**

Reablement was found to be more cost-effective than usual care. The assessments of performance and satisfaction regarding daily activities were significantly higher in the reablement group compared with the control group and this was achieved at lower cost. Importantly too, in the post-trial period, the intervention group requested significantly fewer home visits which were, on average, of significantly shorter duration compared with the control group. Expenditure on home visits was significantly lower for the reablement group.

**Conclusions:**

Reablement is a more cost-effective intervention compared with usual care. Reablement has a potentially large effect on the demand for compensating home-based care services. Policy-makers should therefore consider implementing reablement on a larger scale.

## Background

Disability in older adults involves functional decline and, as such, is an important determinant of long-term care utilization [1]. Thus, interventions that significantly influence people’s disability status can potentially reduce the use of, and expenditure on, home-based care. Reablement, also termed restorative care, is a form of home-based rehabilitation, which focuses on improving independent functioning in daily activities perceived as important by the participant. This is contrary to simply doing tasks for people indefinitely, which has been the traditional way home-based care services have been offered. Other characteristics of reablement are that the intervention is time-limited, person-centered and delivered by integrated teams consisting of various professionals such as occupational therapists, physiotherapists, nurses, auxiliary nurses and assistants. Furthermore, the intervention is implemented in the home setting or in the local community.

Recently, the first randomized controlled trial (RCT) on reablement conducted in Europe demonstrated that a 10-week reablement program significantly improved *self-perceived activity performance* (COPM-P) and *satisfaction with performance* (COPM-S) in community-dwelling older adults in a Norwegian municipality [2]. However, this RCT was not evaluated from an economic point of view.

To date, two economic evaluations of reablement have been published in peer-reviewed journals. The first study was a large retrospective cohort study lasting nearly 5 years including older adults who had received reablement versus conventional home-based care [3]. The results showed that the likelihood of using any type of home-based care service was reduced for 3 years, and the need for personal care service was reduced for nearly 5 years. The reduced use of home-based care services was associated with median cost savings per person of approximately $12,500 Australian dollars (AUD). The second study, also from Australia, was a large RCT with a 2-year follow up [4]. Participants undertaking reablement were found to use fewer home-based care hours and were less likely to be approved for a higher level of care such as nursing home placement. Further, they were less likely to be admitted to emergency departments or to have an unplanned hospital admission. Finally, reablement resulted in lower total home-based care costs than conventional care: $4,096 versus $5,270 AUD for the first year, and $5,833 versus $8,374 AUD for the total 2-year period.

Based on detailed data on home visits combined with data on outcome measures, the objective of this study was to provide an economic evaluation of reablement to complement the above-mentioned effect studies. The economic evaluation had two parts: an analysis of the cost-effectiveness of the intervention based on estimates of the incremental cost-effectiveness ratio (ICER) and estimates of post-trial differences in home-based care expenditure.

In this first cost-effectiveness study on reablement conducted outside Australia, we found a negative incremental cost-effectiveness ratio (ICER), indicating that reablement leads to better outcomes (COPM-P and COPM-S) at a lower cost compared with compensating home-based care services. Importantly too, we also found significant differences in both the number of home visits and the duration of compensating home-based care services in the post-trial period. In summary, reablement led to lower expenditure on compensating home-based care services in the post-trial period. We concluded that reablement could be a promising way forward in the care of community-dwelling older adults.

### Demographic changes and distribution of services

In Norway, there is a view that reablement needs to be central to efforts aimed at meeting the major challenge of financing long-term care. The most recent figures from the Central Bureau of Statistics in Norway (SSB) show that expenses related to care services exceeded 100 billion Norwegian kroner (NOK) in 2014 [5]. The share of home-based care services increased to an all-time high, making up almost half of the total expenses, while the share of expenses related to long-term and short-term care at institutions decreased to 45 %. Five per cent of the total expenses covered social activity and additional services.

Similarly, in Norway, the total number of users of home-based care services, that is, nursing services in the home, home help, rehabilitation and other services for users living at home, independent of age and diagnosis, has remained almost unchanged at 190,000 users. The number of older users (67 years and over) has decreased by 1,000 to a total of 107,500, and the number of young users has increased by approximately the same number, to a total of 84,500 users. There has been a marked decrease in the number of users of home-based care services for people aged 80 years and over.

However, as reported by SSB [5], the share of users with extensive need for assistance has increased in all age groups. Since 2007, when registration of needs started, there has been a continuous increase, regardless of age. So, while the total number of users is stable, the increase in users with extensive needs necessitates more resources to be allocated to the sector. One indicator of this development is that the average number of hours of home help or compensating care has increased by 10 % since 2013. In 2014, each user, on average, received 9 h of home help per week. The corresponding hours of home-based nursing is unchanged and is approximately half the level of home help services, that is 4.6 h, on average, per user per week [5].

If reablement can postpone individuals’ need for compensating care or reduce the level of need for compensating care, a continuous increase in the growth of users with extensive needs, is not inevitable. Rather, it may be (partly at least) endogenously determined, that is, dependent on the allocation of resources to reablement and other cost-effective preventive interventions by local authorities. This cost-effectiveness study of reablement is a contribution to increasing the knowledge base related to measures that potentially can make a difference, not only in terms of expenditure levels in the long-term care sector, but also in relation to improved quality of life for individuals.

### Implementation of reablement—a short overview

Reablement has been implemented in countries like the UK [6], US [7], Australia [4] and New Zealand [8] from around the year 2000. A similar development has occurred within the three Scandinavian countries. Although influenced by the same demographic and socio-economic trends in high-income countries, it appears that the evolution of international and Scandinavian reablement has followed two parallel, but separate, paths. The implementation of reablement in Scandinavia started in the municipality of Östersund in 1999 and spread from there to other municipalities in Sweden. The development in Sweden inspired Denmark to get started in 2007, in particular in the municipality of Fredericia [9, 10]. Nowadays, almost all Danish municipalities have started offering reablement services [11]. Since the first municipalities started implementing reablement in 2012, there has also been a rapid development in Norway [12]. To date, 34 % of all Norwegian municipalities are offering reablement services and the growth continues.

In the Norwegian municipality we studied, the researchers initiated the implementation of reablement. The municipality agreed to participate in the study because of a need for more services that encourage more activity in older adults. In addition, there was a need for sustainable services in long-term care (LCT). Finally, the municipality agreed to participate due to the support offered during the implementation of the intervention.

## Methods

### Design and data collection

A parallel-group randomized controlled superiority trial was conducted, in which all participants were assessed at baseline, and after 3 and 9 months. The study was conducted in a primary care setting in a rural municipality in Western Norway with approximately 14,000 inhabitants. The recruitment period lasted from May 2012 until February 2014. Data collection was terminated in December 2014. Older adults living in the municipality were randomized to receive either reablement or usual care. The intervention period lasted on average 10 weeks. The study met the terms of the CONSORT statement for transparent reporting and was registered on November 20, 2012 in ClinicalTrials.gov, identifier NCT02043262. Ethics approval for the study was granted by the Regional Committees for Medical and Health Research Ethics in Norway (REK West, 2012/295). All participants received information about the study and gave written consent. Procedures were conducted in accordance with the Declaration of Helsinki.

The methods used in the effect study have been described in detail in the study protocol and in the published results of the health effects [2, 13]. People applying for, or referred for, home-based care services were potential participants for the study based on their self-reported activity limitations. Some of the participants had been hospitalized due to an acute illness, while others were recruited after having gradually developed functional decline not needing hospitalization or institution-based treatment. Home-dwelling people over the age of 18 years, who lived in the municipality, were able to understand Norwegian, and had some functional decline in one or more daily activities were included. We excluded people if they were in need of institution-based rehabilitation or a nursing home placement, were terminally ill, or were moderately or severely cognitively reduced.

A biostatistician not involved in the assignment of participants to groups, performed the randomization with an allocation ratio of 1:1, using a computer-generated permuted block randomization sequence, with randomly selected block sizes of lengths 2 and 4. The allocation sequence was concealed in sequentially numbered, opaque, sealed envelopes. The allocation list was stored in a safe deposit box in a central office in the municipality. Neither research assistants nor health-care providers enrolling participants had influence on group allocation. The research assistants performed the baseline assessments in the participant’s home before randomization. The participants were asked not to reveal their group allocation to the research assistants during follow-up assessments. The success of the research assistants’ blinding was registered. Researchers handling data entry and data analysis were blinded to group allocation.

The reablement intervention consisted of both general and individual features. Among the general features was a maximum rehabilitation period of 3 months. Further, as part of the baseline assessment, the occupational therapist and physiotherapist used the instrument Canadian Occupational Performance Measure (COPM) to identify activity limitations perceived as important by the participant [14]. During a semi-structured interview, the participant was encouraged to identify problems with his/her self-care, productivity and leisure activities. The participant rated the importance of each identified activity, and prioritized and rated the five most important activities in performance (COPM-P) and satisfaction with performance (COPM-S) again on 1 to 10-point scales. Further, we collected socio-demographic characteristics at baseline.

The therapists supervised the home-based care personnel, some of whom had no formal education (assistants), in how to encourage and assist the person in the daily training. The focus was on stimulating the participants to perform the daily activities themselves, rather than letting others do it for them. Of the individual features of the intervention, we mention here (i) training in daily activities such as dressing, bus transport, vacuuming, (ii) advice on appropriate assistive technology or adapting the activity itself or the environment, in order to simplify activity performance and (iii) exercises incorporated into daily routines, like indoor or outdoor walking with or without walking aids, climbing stairs, performing exercises to improve strength, balance or fine motor skills.

The control intervention was usual care. The provision of usual care was not limited to 3 months and could continue beyond the reablement intervention period for both groups of participants. For most of the participants, usual care meant receiving the type of services offered to homebound people in the majority of municipalities in Norway. Usual care often comprises compensating help and its content is delivered according to the needs described in applications made by the participants. This may be personal or practical assistance, meals on wheels, safety alarm, or assistive technology. However, it may also involve rehabilitation efforts by health professionals such as occupational therapists and physiotherapists.

Another important input in the economic evaluation was cost data. The cost data used in this study were based on registrations of the number of home visits to each individual participant; the duration of the visits; the type of professional delivering the care and wage costs differentiated according to profession. Such registrations were made over a period of 9 months. A folder containing specially designed forms was kept in each participant’s home. Every time the healthcare provider(s) came to visit the participant, they had to register their name, date, healthcare profession, tasks performed and minutes spent working (in 5-min sequences) in the folder. Travel time and time the healthcare providers spent working in their offices, were not included. Based on data on hourly wages including payroll taxes (for the different categories of healthcare professionals), we calculated individual home-based care expenditure and expenditure per group.

The sample used in the effect study consisted of 61 participants randomized to either reablement (*n =* 31) or to usual care (*n =* 30). Here the drop-out rate was 11 % and 16 % at the 3-month and 9-month follow ups respectively, the majority being caused by mortalities among participants. However, there were only registration data for 29 and 23 participants in the intervention group and control group respectively, giving a registration drop-out rate of 15 %. Reasons for missing registrations were registrations forgotten (*n =* 3), registrations lost (*n =* 2), participant withdrawal before the intervention commenced (*n =* 2), and no home-based services delivered (*n =* 2). The final sample was further reduced due to the requirement that we needed to calculate each participant’s incremental cost-effectiveness ratio. Hence, the sample used in the cost-effectiveness analysis comprised only the participants who were included in both the two prior samples, which included 25 participants in the intervention group and 21 participants in the control group, giving a 25 % drop-out rate from the original sample of 61 participants.

### Statistical method

All methods of economic evaluation involve some kind of comparison between alternative interventions, treatments or programs. A cost-benefit approach should, in principle, address allocative efficiency, that is, contribute to the decision-making process of how many resources should be allocated to, for instance, health, education or long-term care. A cost-effectiveness analysis, on the other hand, addresses the question of productive efficiency in the process of providing a specific service at the lowest possible cost. In the context of home-based care services, this approach seemed to be the relevant approach focusing on the incremental cost-effectiveness ratio (ICER). This approach, though, is not without its limitations. The limitations are related to interpretation of point estimates and, in particular, to uncertainty. In a stochastic analysis, as is the case with the current study, we had to address uncertainty due to sampling variation, an issue of particular importance as the sample was small. Based on a well-established analytical framework [15], we proceeded as follow: Mean costs and effects in both groups were estimated and reported along with standard deviations. Furthermore, *mean differences* and standard deviation in costs and effects were reported. These latter estimates were combined to obtain estimates of the incremental cost-effectiveness ratios (ICERs). Based on the standard errors, the point estimates of the ICERs were estimated. As discussed below, confidence intervals around the point estimates of the ICERs were of limited use. Therefore, we needed a different approach to determine the uncertainty around the point estimates.

Bootstrapping has been used on cost-effectiveness data previously and is well-documented [16–19]. We followed the original sampling procedure. We estimated the uncertainty around the cost-effectiveness estimates using bootstrap, which meant the observed sample was treated as a proxy for the population of community-dwelling older adults and resampled (4000 times) from that sample to estimate empirically the distribution around the means. However, the point estimates of the ICERs were negative and negative ratios pose problems when estimating confidence intervals. We needed to apply a method that did not treat negative ratios in the same way whether they be caused by greater costs and smaller effects (north-west quadrant of the cost-effectiveness plane) or by lower costs and greater effects (south-east quadrant, as we found). Our solution was to discuss uncertainty in cost-effectiveness by means of the cost-effectiveness acceptability curve (CEAC) based on the bootstrap data, a technique first applied by Van Hout et al. [20].

## Results

### Baseline characteristics

Table 1 presents the baseline demographic characteristics by study groups. Overall, the groups were similar at baseline with one exception: the control group scored significantly higher on COPM-S at baseline compared with that of the intervention group (3.7 vs 2.6 points respectively). As in the effect study, the sample consisted of predominantly older females (72 %), who lived alone (79 %) and had no higher education (82 %). The number of mortalities and the number of prescriptions indicated a frail sample.

**Table 1:** Baseline characteristics

Characteristics	Intervention, *n =* 25	Control, *n =* 21	*P*-Value
Age, mean years ± SD, range	79.9 ± 10.4, 45	78.1 ± 10.4, 42	.57
Female, no (%)	19 (76.0)	14 (66.7)	.53
Married/cohabiting, no (%)	7 (28.0)	2 (9.5)	.12
Education < university/university college, no (%)	23 (92.0)	17 (81.0)	.27
Retired, no (%)	23 (92.0)	18 (85.7)	.32
Motivation for rehabilitation, scale 1–10, 10 is best, mean ± SD	7.6 ± 2.3	7.7 ± 2.0	.84
Total number of prescribed medications, mean ± SD, range	6.2 ± 3.0, 13	6.4 ± 3.3, 10	.81
Self-reported number of medical conditions, mean ± SD, range	3.1 ± 1.7, 8	3.0 ± 1.2, 4	.79
Activity performance (COPM-P), sum score, scale 1–10, 10 is best, mean ± SD	2.5 ± 1.5	2.7 ± 1.4	.63
Activity satisfaction (COPM-S), sum score, scale 1–10, 10 is best, mean ± SD	2.6 ± 1.6	3.7 ± 1.9	.04

**Table 2:** Difference in mean change of COPM-P and COPM-S points per group. Intervention period. Two-sample t-test with equal variances

	Group	*N*	Mean (SD)	*p*-value
COPM-P				.043
	Intervention	25	4.33 (2.59)	
	Control	21	3.01 (2.48)	
	Difference		1.32	
COPM-S				.030
	Intervention	24	4.30 (2.40)	
	Control	21	2.58 (2.67)	
	Difference		1.72	

### Mean effect and cost estimates

We found significant changes in both mean COPM-P and COPM-S points after 3 months, as reported in Table 2. Based on a two sample *t*-test, the difference in mean change in COPM-P was 1.32 points, somewhat lower than the effect reported in the effect study but still significant and in the same range. However, while the effect study did not report significant changes in COPM-S after 3 months, we found a significant change of 1.72 points.

Turning to the cost measure, we found a significant difference in both the number of visits and the duration of visits during the intervention period. While the intervention group was visited fewer times (Table 3, column 1) than the control group on average, the mean duration of visits was approximately one minute longer (not reported in the table). The higher expenditure per visit for the intervention group (column 2) reflects higher competence and subsequently higher wages for staff involved in reablement compared with staff involved in usual care. The significant difference in costs per visit was rather modest though (NOK 9.61), so, in total, the aggregate cost during the intervention period was approximately 12,000 NOK *lower* for the intervention group. The lower number of visits received by the intervention group, a somewhat unexpected result, explains the cost difference. The difference in mean cost per participant between the two groups was not significant based on per participant estimations (column 5). Nevertheless, we used these mean estimates in the ICER calculation rather than the calculated average cost per participant (column 4). Doing this, we used a more ‘conservative’ cost difference compared with the difference reported in column 4.

**Table 3:** Differences in mean number of visits, mean costs per visit, mean cost per participant. Intervention period. Two-sample t-tests with unequal variances. Cost figures in NOK

		(1)	(2)	(3)	(4)		(5)	(6)
Group	No. of visits V	Average number of visits	Mean cost per visit (SD)	Total costs (2)xV	Average cost (3)/N	No. of participant *N*	Mean cost per partic. (SD)	Total costs
Intervention	1624	65	97.49 (82.85)	158323.76	6332.95	25	6322.78 (4101.98)	158069.50
Control	1806	86	87.88 (76.60)	158711.28	7557.68	21	7456.77 (12952.97)	156592.17
Difference		21	9.61	−387.52	−1224.72		−1134.00	1477.33
*p*-value			.00				.68	

As summarized in Table 4, the difference in mean costs per participant was approximately 1,134 NOK, that is, the mean costs per participant were lower for the intervention group compared with the control group at 3 months follow up. In Table 4, ΔC denotes the change in costs between baseline and the 3-month follow-up registrations, while ΔE_P_ and ΔE_S_ denote the changes in COPM-P and COPM-S, respectively. The differences or changes in mean effects are 1.32 and 1.72 points for COPM-P and COPM-S, in favour of the intervention group.

**Table 4:** Summary statistics (intervention period). Cost figures in NOK

	Control group (CG) (*n =* 21)			Intervention group (IG) (*n =* 25)			Difference		
	Cost (NOK)	Effect (COPM-P)	Effect (COPM-S)	Cost (NOK)	Effect (COPM-P)	Effect (COPM-S)	ΔC	ΔE_P_	ΔE_S_
Mean	7456.77	3.01	2.58	6322.78	4.33	4.30	−1134.0	1.32	1.72
Standard deviation (SD)	12952.97	2.48	2.67	4101.98	2.59	2.40			
Standard error (SE)	2826.57	0.54	0.58	820.40	0.52	0.50	2065.59	1.09	1.03

Based on the results in Table 4, we calculated the incremental cost-effectiveness ratios (ICER) defined by the general expression ICER = ΔC/ΔE. In conclusion, the expenditure needed to increase COPM-P by one point was 868.18 NOK (ICER_COPM-P_ = − 868.18) lower on average for the intervention group compared with the control group. Concerning COPM-S, the expenditure needed to increase COPM-S by one point was 666.30 NOK lower on average for the intervention group compared with the control group (ICER_COPM-S_ = − 666.30). In both cases, we found a combination of differences in costs and differences in effects located in the south-east quadrant of the cost-effectiveness plane, that is, reablement was in each case more cost-efficient compared with usual care.

**Fig. 1:**
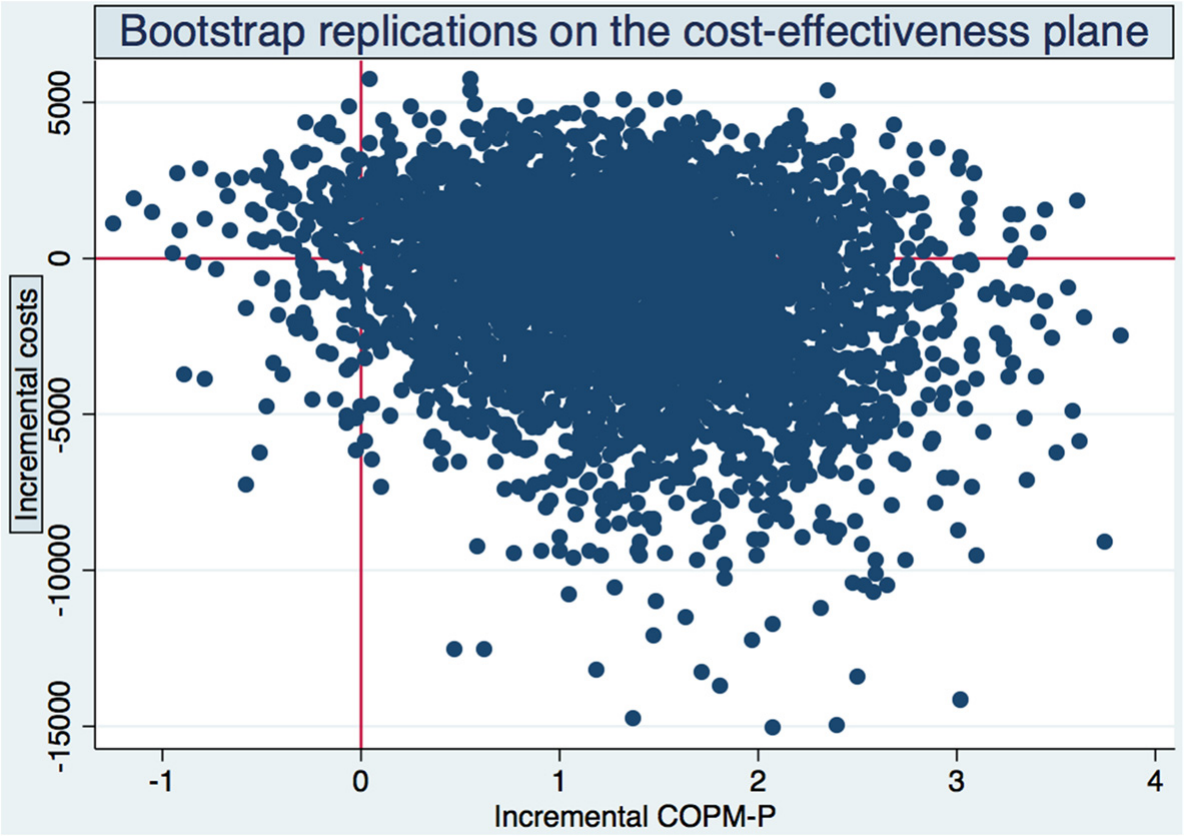
Bootstrap replications of the cost-effectiveness plane. COPM-P

**Fig. 2:**
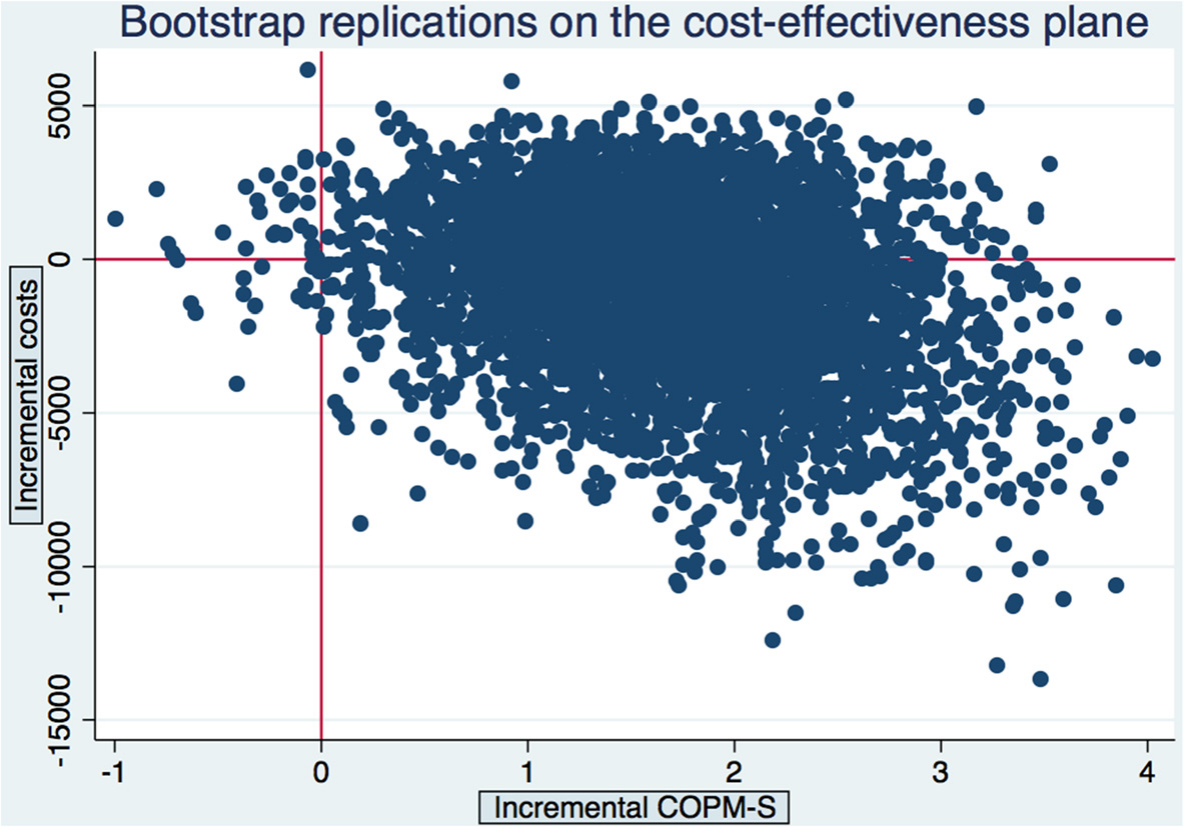
Bootstrap replications of the cost-effectiveness plane. COPM-S

However, it is not sufficient to report the point estimates of cost-effectiveness ratios. Our main concern was uncertainty due to sampling variation. Thus, we needed to approach the issue of uncertainty surrounding these estimates using a bootstrap procedure. Figure 1 shows the bootstrap replications on the cost-effectiveness plane using difference in COPM-P as effect variable, while Fig. 2 shows the replications using COPM-S as effect variable.

It can be seen in the north-west (NW) quadrant of the cost-effectiveness plane (higher costs, lower effect) that reablement is less favourable than usual care, while in the south-east (SE) quadrant (lower costs, higher effect), reablement is favourable. In both cases, the ICERs are negative. Likewise, cost-effectiveness ratios in the north-east quadrant (NE) and south-west (SW) quadrant were positive but for different reasons. In short, confidence intervals for the ICERs were not informative.

The bootstrap procedure gave us an approximation of the cost-effect joint densities (Figs. 1 and 2). On the basis of these joint densities, we calculated the probability that reablement is actually cost-effective. The so-called Cost-Effectiveness Acceptability Curve (CEAC) [20] is constructed by imagining a decision-maker changing the ceiling ratio, for example, willingness to pay, from (i) no willingness to pay for COPM-P or COPM-S gains to (ii) a willingness to pay for positive infinity for such gains. In the first case, the line representing the willingness to pay is horizontal and lies on the x-axis. Reablement is cost-effective only if its costs are less than usual care. In the second case, the line representing the willingness to pay is vertical and lies on the y-axis. In that case, costs are of no concern and reablement is chosen if it is more effective compared with usual care.

We did not plot the CEACs here but calculations on the basis of the joint density of ICER COPM-P showed that approximately 62 % (2470/4000) of the bootstrap replications fell in the SE quadrant. In other words, we found indications that there was 62 % probability that reablement was cost-effective if the decision-maker’s willingness to pay (WTP) for COPM-P gain was zero. If the WTP was approximately equal to our point estimate (870 NOK per COPM-P point), there was a 76 % probability that reablement was cost-effective. If WTP was, for instance, 2000 NOK per COPM-P point, there was an 88 % probability that reablement was cost-effective.

In the case of COPM-S, we found indications that there was 60 % probability that reablement was cost-effective if the decision-maker’s willingness to pay for COPM-S gain was zero; a 73 % probability if the WTP was 667 NOK per COPM-S gain and a 91 % probability if WTP equalled 2000 NOK.

### Differences in expenditure and effects between 3 and 9 months follow up

Because reablement addresses independent performance in daily activities perceived as important by the participants, our hypothesis was that reablement would reduce long-term care expenditure by diminishing the need for compensating home-based care services after the trial period. Indeed, our analysis showed a significant difference in the use of such services in the 6-month period following the trial (Table 5).

**Table 5:** Mean costs per person per group and total costs per group. Mean cost per visit, mean number of visits, total and average costs. Post-intervention period (6 months). Two-sample t-tests with unequal variances

		(1)	(2)	(3)	(4)		(5)	(6)
Group	No. of visits	Average number of visits	Mean cost per visit (SD)	Total costs	Average cost	No. of partic.	Mean cost per person (SD)	Total costs
Intervention	2202	88	73.50 (65.63)	161847.00	6473.88	25	6470.82 (10559.00)	161770.50
Control	3318	158	88.18 (81.08)	292581.24	13932.44	21	13914.31 (28962.05)	292200.51
Difference		−78	−14.68	−130734.24	7458.56		−7443.23	−130430.01
*p*-value			.00				.24	

The intervention group received on average 88 home visits compared with the control group receiving 158 visits on average, a difference of 78 visits on average. We also found a significant difference in average duration per visit of 1.30 min (not reported in the table). The aggregate difference in expenditure in the 6-month period was approximately 130,000 NOK (column 3). Thus, the average cost difference per participant was approximately 7500 NOK (column 4).

The estimated direct cost difference per participant was not significant (column 5). We attribute the non-significant result to the small number of observations (46 participants). The direction and size of the differences are in line with the estimated results based on visits. The mean cost per person in the intervention group was 6,470 NOK, while the average in the control group was 13,914 NOK. The aggregate difference in total cost was approximately 130,000 NOK based on these estimates (column 6).

We also found a significant lasting difference of averages in COPM-P and COPM-S, that is, significant differences between baseline and 9 months follow up (not reported in the table). A significant difference between baseline and 3-month follow up was sustained in both measures but the major difference was achieved during the intervention period. In fact, looking at the changes between the 3-month and 9-month follow up, the difference between the groups was not significant.

## Discussion

### Implications for policymakers

In this cost-effectiveness study, reablement was found to be more cost-effective than usual care at 3 months follow up both in terms of improved performance in, and satisfaction with, daily activities. Moreover, the results of this study demonstrate that reablement is associated with significantly lower demand for traditional compensating care in the 6-month period following the intervention, and consequently, lower average expenditures for the intervention group compared with the control group. Cost savings were also found in the two other economic evaluations of reablement [3, 4]. These studies were substantially larger in terms of participants and used longer follow-up periods than the present study. Although the long-term effects are not studied here due to lack of data, the Australian studies point in the direction that the significant effects of reablement can be traced two years after the intervention [4] and even five years after [3]. Consequently, policy-makers need to consider implementing reablement on a larger scale. Nevertheless, this recommendation comes with a few words of caution.

Reablement is a client-centred intervention, where the older adults decide themselves which activities shall be the goals for their rehabilitation. This means that the older adults can prioritize other activities, beyond those that lead to a reduced need for home-based services. This happened often in the current study. The older adults did, for instance, prioritize outdoor physical activities, leisure activities or social activities, none of which led to reduced need for home-based care services.

In an effort to maximise the benefits gained, policy-makers might be tempted by the results in the current study to narrow the older adults’ choices to only activities that have the potential to reduce compensating help. We strongly discourage such a strategy. On the contrary, the major success criteria of reablement in the current study were that we used the Canadian Occupational Performance Measure instrument during the baseline interview. Using a client-centred goal formulation structure like COPM improves participation [21] and results [22], while a staff-directed goal-setting and rehabilitation process reduces participant engagement [23]. The motivational COPM interview allowed older adults to identify problems in their self-care, productivity and leisure activities and prioritize the most important of them. The effect was that the older adults were motivated and stimulated to work hard towards reaching their goals. This strong motivation and active engagement would have been less likely if activity choices were restricted to personal care and domestic chores.

### Implications for lifetime healthcare expenditures

As pointed out in the introduction, interventions that significantly influence people’s disability status can potentially reduce the use of home-based care services and thereby reduce (the growth rate of) expenditure. As shown, reablement has this potential and we find interesting connections between our research and the larger issue of healthy ageing.

Healthy ageing, a scenario in which the population in a society experiences on average increased years lived in good health and increased life expectancy, implies that the increase in life expectancy is ‘translated’ into an increase in number of years spent in good health, leaving the demand for healthcare unchanged. As a result, the increase in average age in a population does not significantly influence healthcare expenditure per capita. The (inevitable) individual healthcare costs are merely postponed. Some empirical studies give support to the healthy ageing scenario: individual healthcare costs increase substantially when an individual is close to death and these costs constitute a major part of the lifetime healthcare expenditures [24, 25]. These studies suggest that demographic change *per se* will not have a large impact on future aggregate healthcare expenditure.

According to van Baal and Wong [26], it is not surprising that individual variation in healthcare expenditure to a large extent can be explained by time to death (TTD) rather than time from birth (age). They argue that we should consider age and TTD as proxy variables for morbidity and disability, which are the most important drivers of individual healthcare demand. De Meijer et al. [1] support this argument. Of particular relevance here, they found that *age* significantly determines *home-based care use and expenditure* but the effect is substantially reduced when controlling for *disability*. Furthermore, the effect of disability has a much more sizeable effect on home-based care use than TTD.

The results reported by De Meijer et al. [1] also imply that disability status is one of the key variables in terms of deciding individual demand for long-term care. Age and disability proved to be the main determinants of utilization. Interestingly, the presence of at least one disability displayed a greater effect on utilization than any additional disabilities. By contrast, general health status hardly affects long-term care use.

Crimmins et al. [27] emphasize that most of our knowledge about trends in disability is based on changes in the prevalence of disability. However, understanding the dynamic forces affecting the prevalence of disability is important in interpreting changes in prevalence and to make better projections of the likelihood of continued change. Crimmins et al. [27] point to several dynamic processes including postponed onset of disability; increase in recovery and/or changes in mortality of the disabled and nondisabled. As we have shown, reablement has the potential to achieve a recovery for older adults.

### Strengths and limitations

The strengths and limitations of this study are the same as in the effectiveness study [2]. However, in addition, the major strength of the current economic study is that we use detailed costs and outcome data to evaluate the cost-effectiveness of reablement. A limitation of the cost-effectiveness study is, however, a rather large drop-out rate (25 %) in the final sample. However, the bootstrap replications improved the estimate of the ICER sampling distribution. Another limitation is that only face-to-face time spent in the participants’ homes were included in the time registration, not time spent in the healthcare providers’ offices. It is possible that reablement requires more time for collaboration and registration than usual care. Further, a longer follow-up than 6 months would have been preferable in order to evaluate the sustainability of the reduction in expenditure.

## Conclusions

To the best of our knowledge, this is the first published cost-effectiveness study on reablement conducted outside Australia. We conclude that reablement stands out as a promising intervention, not only because it seems to decrease expenditure, but also because older adults feel they improve their performance and satisfaction in daily life activities. The combination of lower costs and higher effects is the kind of policy measure that will be of interest to policy-makers.

## Abbreviations

AUD: Australian dollar
CEAC: cost-effectiveness acceptability curve
COPM: Canadian occupational performance measure
ICER: incremental cost-effectiveness ratio
HCE: health care expenditures
NOK: Norwegian crowns
RCT: randomized controlled trial
TTD: time to death
WTP: willingness to pay
